# Supplementary material for: Functional traits and phenotypic plasticity modulate species coexistence across contrasting climatic conditions
Source: Nat Commun. 2019 Jun 11;10:2555. doi: 10.1038/s41467-019-10453-0 (PMC6560116; doi:10.1038/s41467-019-10453-0)

SUPPLEMENTARY INFORMATION

TITLE: Functional traits and phenotypic plasticity modulate species coexistence across contrasting climatic conditions

FIRST AUTHOR: Pérez-Ramos et al.

Supplementary Table 1. Mean ± standard-error values of the functional traits quantified in this study on the ten study species, for the two climatic treatments. Significant differences between treatments have been highlighted in bold letters and asterisks. Seed mass was not included because we used the same seed pool for both climatic treatments. Maximum leaf-level photosynthesis and light response curves could not be accurately measured for *Capsella bursa-pastoris* due to the reduced size and fragility of their leaves. The level of significance has been indicated with asterisks (*** p<0.001; ** p<0.01; * p<0.05), according to ANOVA-*one way* tests.

		Borago officinalis		*Bromus madritensis*		*Calendula arvensis*		*Capsella bursa-pastoris*		*Diplotaxis erucoides*	
	Family	Boraginaceae		Poaceae		Asteraceae		Brassicaceae		Brassicaceae	
	Trait	CONTROL	DROUGHT	CONTROL	DROUGHT	CONTROL	DROUGHT	CONTROL	DROUGHT	CONTROL	DROUGHT
WHOLE-PLANT TRAITS	Plant Height (cm)	57.4 ± 2.8	**30.8 ± 4.7*** **	62.3 ± 3.1	**18.6 ± 7.3*** **	36.3 ± 1.5	**10.8 ± 1.8*** **	12.9 ± 1.9	2 ± 16.3	50.8 ± 1.5	**18.7 ± 1.8*** **
	Plant Volume (m^3)	0.2 ± 0.02	0.021 ± 0.08	0.04 ±0.05	0.004 ± 0.12	0.2 ± 0.02	**0.003 ± 0.03*** **	0.001 ±0.03	0.00001 ± 0.26	0.3 ± 0.02	**0.005 ± 0.03*** **
MORPHOLOGICAL TRAITS	Leaf size (cm^2)	14.0 ± 1.9	11.5 ± 5.5	2.7 ± 2.1	6.1 ± 3.2	5.6 ± 1.0	2.1 ± 1.2	2.3 ± 1.3	1.2 ± 10.9	16.1 ± 1.0	**10.1 ± 1.2***
	Specific Leaf Area (cm^2 g^{-1})	234.3 ± 12.9	213.7 ± 37.2	295.2 ± 14.1	249.0 ± 21.5	264.0 ± 6.8	300.8 ± 8.4	183.9 ± 8.8	171.7 ± 74.4	185.8 ± 6.8	22.8 ± 8.2
	Leaf Dry Matter Content (mg g^{-1})	104.2 ± 46.6	427.8 ± 133.9	215.7 ± 50.6	212.0 ± 77.3	103.6 ± 24.4	93.0 ± 30.1	208.0 ± 31.8	250.9 ± 267.8	146.0 ± 24.4	136.8 ± 30.3
	Root Diameter (mm)	0.42 ± 0.07	**1.00 ± 0.06*** **	0.19 ± 0.05	0.18 ± 0.05	0.30 ± 0.06	0.33 ± 0.05	0.20 ± 0.05	**0.68 ± 0.07*** **	0.27 ± 0.04	0.43 ± 0.05
	Specific Root Area (cm^2 g^{-1})	15.3 ± 3.7	9.8 ± 3.2	33.1 ± 2.9	35.6 ± 2.4	26.5 ± 3.2	15.3 ± 2.9	8.0 ± 2.6	12.1 ± 3.7	26.7 ± 2.3	**8.6 ± 2.9*** **
	Root Density (g cm^{-3})	0.75 ± 0.33	0.44 ± 0.28	0.68 ± 0.25	0.67 ± 0.22	0.51 ± 0.28	0.81 ± 0.25	3.24 ± 0.23	**0.50 ± 0.33*** **	0.61 ± 0.20	1.16 ± 0.25
PHYSIOLOGICAL LEAF TRAITS	Max. Photosynthesis (moles m^{-2} s^{-1})	21.4 ± 1.7	**5.1 ± 1.5***	2.4 ± 0.7	9.7 ± 0.6	13.9 ± 1.3	17.6 ± 3.0			18.6 ± 2.7	23.5 ± 2.6
	Stomatal Conductance (moles m^{-2} s^{-1})	0.49 ± 0.08	0.08 ± 0.02	0.03 ± 0.01	0.15 ± 0.03	0.22 ± 0.03	0.72 ± 0.21			0.30 ± 0.07	**1.04 ± 0.21*** **
	Convexity	0.53 ± 0.06	0.56 ± 0.28	0.87 ± 0.05	0.23 ± 0.23	0.17 ± 0.06	0.56 ± 0.23			0.86 ± 0.07	0.53 ± 0.27
	Light Compensation Point	35.8 ± 10.3	34.3 ± 11.9	40.1 ± 11.2	55.2 ± 92.8	13.0 ± 1.1	35.8 ± 16.8			24.5 ± 3.3	42.0 ± 7.6
	Light Saturation Point	465.3 ± 76.4	171.3 ± 16.7	161.7 ± 18.2	214.7 ± 217.3	185.5 ± 51.8	546.0 ± 75.6			231.0 ± 40.3	604.3 ± 47.7
	Leaf Nitrogen Content (mg g^{-1})	19.1 ± 0.3	**34.3 ± 0.2*** **	18.4 ± 0.4	**30.8 ± 0.1*** **	27.4 ± 0.8	25.5 ± 0.2	21.4 ± 0.1	17.4 ± 0.1	34.1 ± 2.0	**26.7 ± 1.4*** **
	Leaf Carbon Content (mg g^{-1})	371.8 ± 1.2	**396.1 ± 0.4*** **	435.7 ± 0.6	445.5 ± 1.0	404.9 ± 2.2	407.0 ± 0.6	389.5 ± 1.2	397.3 ± 1.0	395.8 ± 2.6	**406.0 ± 5.2***
	Carbon Isotope Ratio	-28.7 ± 0.1	-28.5 ± 0.05	-29.0 ± 0.1	-28.9 ± 0.05	-30.5 ± 0.1	-30.2 ± 0.1	-29.1 ± 0.04	-29.2 ± 0.05	-30.4 ± 0.1	-30.3 ± 0.1
	Nitrogen Isotope Ratio	3.6 ± 0.08	**4.1 ± 0.08***	3.0 ± 0.08	2.5 ± 0.1	6.7 ± 0.1	**5.1 ± 0.1*** **	6.7 ± 0.1	**8.8 ± 0.1*** **	8.2 ± 0.08	7.8 ± 0.08
REPRODUCTIVE TRAITS	Peak of productivity (days)	122.0 ± 0.8	**140.1 ± 3.3** **	120.8 ± 1.6	**143.9 ± 1.4*** **	117.0 ± 5.1	**144.1 ± 1.7*** **	118.1 ± 5.1	**140.7 ± 3.7*** **	117.0 ± 4.2	**144.2 ± 1.5*** **

Supplementary Table 1. Continued.

	Family	*Matricaria chamomilla* Asteraceae		*Medicago polymorpha* Fabaceae		*Papaver rhoeas* Papaveraceae		*Sinapis alba* Brassicaceae		*Vicia sativa* Fabaceae	
	**Trait**	CONTROL	DROUGHT	CONTROL	DROUGHT	CONTROL	DROUGHT	CONTROL	DROUGHT	CONTROL	DROUGHT
WHOLE-PLANT TRAITS	Plant Height (cm)	51.4 ± 2.0	**30.5 ± 4.2****	40.4 ± 1.8	**17.1 ± 4.7*****	62.1 ± 2.5	**35.6 ± 4.7*****	60.7 ± 1.5	**16.8 ± 1.7*****	59.2 ± 2.0	**23.6 ± 4.7*****
	Plant Volume (m^3)	0.1 ± 0.03	0.01 ± 0.07	0.2 ± 0.02	0.012 ± 0.08	0.1 ± 0.04	0.017 ± 0.08	0.3 ± 0.02	**0.006 ± 0.03*****	0.2 ± 0.02	0.039 ± 0.08
MORPHOLOGICAL TRAITS	Leaf size (cm^2)	3.8 ± 1.3	1.4 ± 3.0	6.2 ± 1.2	6.2 ± 3.2	3.8 ± 1.3	0.8 ± 3.2	8.4 ± 1.0	**1.1 ± 1.2*****	7.7 ± 1.3	5.7 ± 3.2
	Specific Leaf Area (cm^2 g^{-1})	174.8 ± 9.1	248.3 ± 20.6	279.7 ± 8.1	277.4 ± 21.5	216.4 ± 11.5	298.4 ± 21.5	221.4 ± 7.0	**159.5 ± 7.9*****	199.5 ± 9.1	178.4 ± 21.5
	Leaf Dry Matter Content (mg g^{-1})	175.6 ± 33.2	149.5 ± 74.3	214.3 ± 29.0	192.9 ± 77.3	170.7 ± 41.3	176.5 ± 77.3	140.1 ± 25.1	**413.8 ± 28.4*****	160.0 ± 32.7	203.8 ± 77.3
	Root Diameter (mm)	0.31 ± 0.05	0.30 ± 0.05	0.39 ± 0.05	0.41 ± 0.07	0.33 ± 0.09	0.84 ± 0.12	0.29 ± 0.05	**0.78 ± 0.05*****	0.35 ± 0.05	0.56 ± 0.05
	Specific Root Area (cm^2 g^{-1})	18.5 ± 2.9	20.6 ± 2.9	14.7 ± 2.9	15.0 ± 3.7	35.1 ± 4.5	12.5 ± 6.4	19.9 ± 2.4	**5.5 ± 2.9***	17.2 ± 2.9	11.5 ± 2.9
	Root Density (g cm^{-3})	0.82± 0.25	0.77 ± 0.25	0.83 ± 0.25	0.68 ± 0.33	0.54 ± 0.40	0.38 ± 0.57	0.72 ± 0.22	1.01 ± 0.25	0.69 ± 0.25	0.64 ± 0.22
PHYSIOLOGICAL LEAF TRAITS	Max. Photosynthesis (moles m^{-2} s^{-1})	5.1 ± 0.7	10.2 ± 2.4	4.2 ± 1.1	11.4 ± 1.7	12.8 ± 2.6	**33.6 ± 7.8*****	23.3 ± 2.2	26.0 ± 3.0	1.8 ± 0.8	5.3 ± 1.4
	Stomatal Conductance (moles m^{-2} s^{-1})	0.07 ± 0.02	0.15 ± 0.07	0.04 ± 0.02	0.13 ± 0.02	0.25 ± 0.07	0.63 ± 0.19	0.33 ± 0.08	0.80 ± 0.20	0.02 ± 0.01	0.06 ± 0.02
	Convexity	0.58 ± 0.13	0.26 ± 0.16	0.55 ± 0.29	0.63 ± 0.22	0.39 ± 0.19	0.35 ± 0.19	0.64 ± 0.08	0.67 ± 0.19	0.48 ± 0.27	0.42 ± 0.21
	Light Compensation Point	77.3 ± 14.8	110.4 ± 80.2	24.8 ± 1.7	61.7 ± 14.1	16.6 ± 10.8	1.5 ± 18.8	20.0 ± 5.5	48.9 ± 7.1	22.0 ± 3.1	41.6 ± 12.1
	Light Saturation Point	259.7 ± 20.9	326.3 ± 172.1	146.3 ± 15.1	240.7 ± 36.6	314 ± 102.6	344.3 ± 105.6	353.3 ± 78.8	797.3 ± 127.3	90.2 ± 10.7	133.2 ± 27.5
	Leaf Nitrogen Content (mg g^{-1})	24.2 ± 0.1	24.7 ± 0.3	42.3 ± 1.7	39.6 ± 0.2	23.2 ± 0.3	**32.9 ± 0.1*****	42.3 ± 0.7	**28.6 ± 0.4*****	37.3 ± 0.3	**46.9 ± 0.1*****
	Leaf Carbon Content (mg g^{-1})	447.5 ± 1.0	438.7 ± 1.3	468.5 ± 2.7	**450.3 ± 1.1*****	396.4 ± 0.7	**412.5 ± 0.2*****	419.4 ± 1.4	**402.9 ± 1.3*****	452.3 ± 1.0	443.5 ± 0.6
	Carbon Isotope Ratio	-30.4 ± 0.03	-30.4 ± 0.08	-27.0 ± 0.1	**-28.4 ± 0.03*****	-27.8 ± 0.05	**-28.7 ± 0.01*****	-30.8 ± 0.04	-30.9 ± 0.02	-26.2 ± 0.1	**-26.6 ± 0.02****
	Nitrogen Isotope Ratio	3.5 ± 0.1	**4.5 ± 0.08*****	-0.5 ± 0.1	-0.04 ± 0.1	5.9 ± 0.08	5.5 ± 0.08	8.4 ± 0.08	**7.1 ± 0.1*****	-0.6 ± 0.1	-0.4 ± 0.1
REPRODUCTIVE TRAITS	Peak of productivity (days)	121.7 ± 1.7	**144.1 ± 1.7*****	122.5 ± 2.5	**140.6 ± 5.5***	121.4 ± 1.8	**144.8 ± 1.5*****	116.1 ± 6.7	**145.0 ± 2.1*****	121.1 ± 2.2	**144.5 ± 1.6*****

Supplementary Table 2. List of species pairs and triplets predicted to coexist in both climatic treatments. These outcomes are predicted following algebraically solution to find unique positive equilibriums of species abundances for all combination from 2 to 10 species. Besides one triplet under control conditions, we did not find positive equilibrium for all species. For more details, see Matías et al. 2018.

	Control Treatment	Drought Treatment
Species pairs	*Capsella bursa-pastoris & Borago officinalis*	*Matricaria chamomilla & Capsella bursa-pastoris*
	Vicia sativa & Borago officinalis	*Medicago polymorpha & Sinapis alba*
	Bromus madritensis & Papaver rhoeas	
	Calendula arvensis & Sinapis alba	
	Papaver roheas & Matricaria chamomilla	
	Sinapis alba & Medicago polymorpha	
Species triplets	*Bromus madritensis & Matricaria chamomilla & Papaver rhoeas*	None

Supplementary Table 3. Results obtained from BEST model selection procedure indicating which set of traits correlate better with the determinants of species coexistence: stabilizing niche differences and average fitness differences (and its two components, demographic and competitive-response differences). All trait combinations shown are statistically significant at $p<0.05$.

Model rank	Climatic treatment	Rho	N traits	Functional Traits
A. Stabilizing niche differences				
	Control	0.591	6	Reproductive phenology, Plant Volume, SRA, SLA, $\delta^{13}C$, Light compensation point.
	Drought	0.563	8	Plant height, Root diameter, Root density, Leaf size, Leaf nitrogen content, Convexity, Light compensation point, g_s
B. Average fitness differences				
	Control	0.416	8	Reproductive phenology, Plant Volume, SLA, Leaf carbon content, Leaf nitrogen content, Convexity, A_{max}
	Drought	0.520	8	Reproductive phenology, Seed mass, Plant volume, $\delta^{13}C$, $\delta^{15}N$, Leaf nitrogen content, Convexity, Light compensation point, A_{max}
C. Demographic differences				
	Control	0.532	7	Reproductive phenology, SRA, Leaf nitrogen content, Convexity, Light compensation point, g_s, A_{max}
	Drought	0.379	8	Plant volume, Root density, LDMC, Leaf size, $\delta^{13}C$, Leaf nitrogen content, g_s, A_{max}
D. Competitive response differences				
	Control	0.403	6	Seed mass, SRA, SLA, $\delta^{13}C$, Convexity, Light compensation point.
	Drought	0.331	6	LDMC, SLA, Leaf size, Leaf nitrogen content, Light compensation point, g_s

Supplementary Table 4. Results from the Generalized Spearman Rank correlations indicating which set of traits are included in the best multivariate model relating trait plasticity and average fitness differences. We computed species' plasticity to drought for each of the 19 traits as the difference in average trait values between the control and the drought treatment. Bold letters indicate those significant relationships at p<0.05.

	Functional Traits	rho^2	F	P
WHOLE-PLANT TRAITS	**Plant Height**	**0.622**	**5.75**	**0.033**
	Plant Volume	0.016	0.06	0.945
MORPHOLOGICAL TRAITS	Leaf size	0.408	2.42	0.159
	Specific Leaf Area	0.249	1.16	0.368
	**Leaf Dry Matter Content**	**0.495**	**3.84**	**0.041**
	**Root Diameter**	**0.469**	**3.62**	**0.044**
	Specific Root Area	0.021	0.08	0.927
	Root Density	0.124	0.5	0.629
PHYSIOLOGICAL LEAF TRAITS	**Max. Photosynthesis**	**0.565**	**4.07**	**0.04**
	Stomatal Conductance	0.322	1.66	0.257
	**Convexity**	**0.439**	**3.47**	**0.049**
	Light Compensation Point	0.238	1.09	0.386
	Light Saturation Point	0.259	1.22	0.351
	Leaf Nitrogen Content	0.153	0.63	0.56
	Leaf Carbon Content	0.162	0.68	0.538
	Carbon Isotope Ratio	0.001	0.001	0.998
REPRODUCTIVE TRAITS	Reproductive phenology	0.101	0.39	0.689

Supplementary Table 5. Factor loadings from the Principal Component Analysis used to evaluate the level of dependence among the 19 functional traits quantified in this study. The most correlated variables with each of the three selected factors have been highlighted in bold letters. See Table 1 for trait abbreviations.

	Factor 1	Factor 2	Factor 3
Plant Height	-0.52	**0.68**	-0.05
Plant Volume	-0.37	**0.73**	-0.42
Leaf size	-0.15	0.38	-0.58
SLA	-0.05	0.03	**0.48**
LDMC	0.16	-0.53	-0.33
Root diameter	0.39	-0.53	**-0.58**
SRA	-0.46	0.55	**0.48**
Root density	0.45	-0.08	0.19
A_{max}	**0.78**	0.29	-0.22
g_s	**0.91**	0.11	-0.11
Convexity	0.07	0.21	-0.28
Light Comp. Point	0.09	-0.36	**0.59**
Light Sat. Point	**0.93**	0.04	0.01
LNC	-0.36	-0.39	**-0.51**
LCC	**-0.58**	-0.47	0.37
C-13	**-0.68**	-0.42	-0.27
N-15	**0.68**	0.60	-0.08
Peak Productivity	0.51	**-0.75**	0.15
Seed mass	-0.47	-0.40	**-0.47**
Proportion of explained variance (%)	27.32	20.60	13.98
Factor identity	Growth rate	Plant size	Plant economics

Supplementary Table 6. Summary of the Pearson´s correlation analyses testing the linkages among the 19 functional traits quantified in this study. Those relationships that were significant at $p<0.05$ have been highlighted in bold letters. See Table 1 for trait abbreviations.

	Plant Height	Plant Volume	Leaf size	SLA	LDMC	Root Diameter	SRA	Root Density	A_{max}	g_s	Convexity	Light Comp. Point	Light Sat. Point	LNC	LCC	C-13	N-15	Reprod. phenology
Plant Height																		
Plant Volume	**0.64**																	
Leaf size	0.21	**0.59**																
SLA	-0.10	-0.19	-0.30															
LDMC	-0.34	-0.38	-0.09	-0.34														
Root Diameter	-0.46	-0.37	-0.04	-0.18	**0.69**													
SRA	**0.56**	0.29	-0.03	0.21	-0.34	**-0.70**												
Root Density	-0.24	-0.18	-0.02	-0.24	-0.02	-0.21	-0.36											
A_{max}	-0.16	0.05	-0.01	0.06	-0.13	0.30	-0.29	0.11										
g_s	-0.39	-0.20	-0.04	0.02	-0.08	0.27	-0.44	**0.49**	**0.83**									
Convexity	0.19	0.20	0.30	-0.16	0.22	0.00	-0.13	0.26	-0.01	0.06								
Light Comp. Point	-0.28	**-0.47**	-0.24	-0.15	0.09	-0.20	-0.05	0.41	-0.30	-0.17	-0.06							
Light Sat. Point	-0.37	-0.28	-0.17	-0.14	0.10	0.24	-0.43	**0.62**	**0.72**	**0.89**	0.13	0.11						
LNC	-0.20	0.23	0.18	-0.16	0.16	0.23	-0.33	-0.16	-0.12	-0.28	0.00	-0.23	-0.37					
LCC	-0.01	-0.16	-0.36	0.14	0.00	-0.30	0.07	0.11	**-0.61**	**-0.57**	-0.08	0.35	**-0.52**	0.46				
C-13	0.16	-0.02	0.07	0.06	0.08	0.12	-0.01	-0.33	**-0.51**	**-0.49**	-0.15	-0.26	**-0.58**	0.42	0.40			
N-15	0.03	0.21	0.10	-0.15	-0.08	0.05	0.06	0.11	**0.70**	**0.62**	0.11	-0.19	**0.58**	-0.37	**-0.71**	**-0.81**		
Reprod. phenology	**-0.85**	**-0.81**	-0.37	0.09	0.34	**0.49**	-0.47	0.18	0.23	0.40	-0.26	0.37	0.39	0.09	0.05	-0.02	-0.09	
Seed mass	0.05	0.08	0.14	-0.38	0.06	0.15	-0.26	-0.11	-0.39	-0.31	-0.09	-0.14	-0.35	**0.47**	0.24	**0.66**	**-0.55**	0.02

Supplementary Figure 1. Projection of the 19 measured functional traits on the plane defined by the two main axes resulting from the Principal Components Analysis.

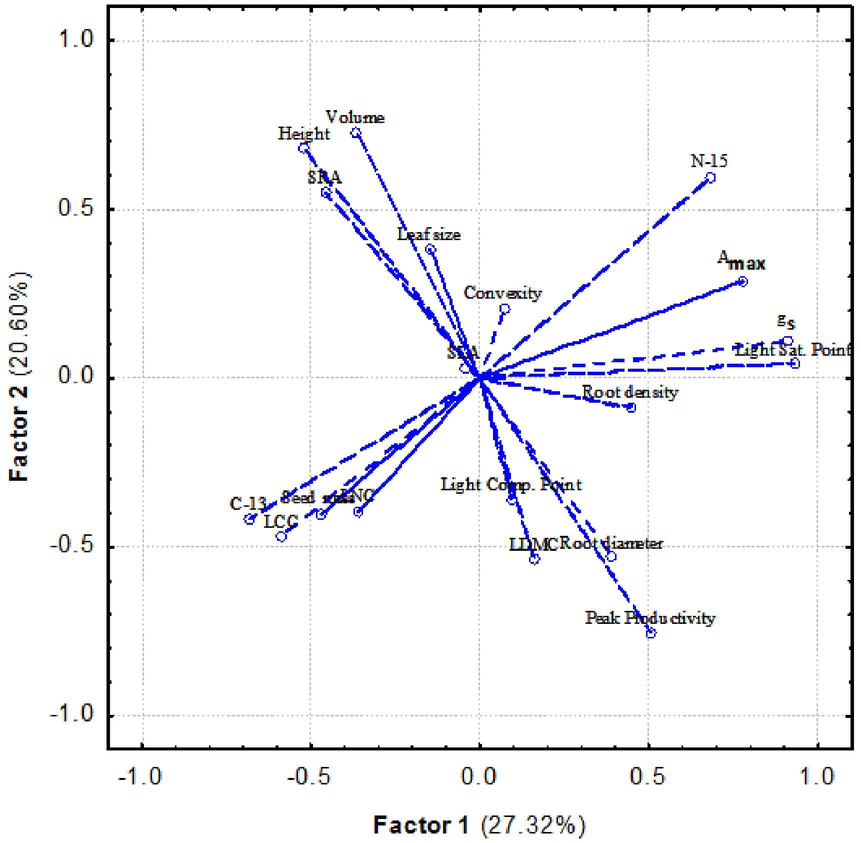

<u>Summary results:</u> The first axis of the PCA (27.32% of the total variance) represented a trade-off between plant growth rate and efficiency in the use of water. Thus, species with the highest growing rates (i.e. with high values of A_{max}, g_s and light saturation point) were the least efficient in the use of water (i.e. with more negative values of $\delta^{13}C$). Additionally, these high-growing species produced less sclerophyllous leaves (i.e. with lower values of LCC) and exhibited higher values of δ-^{15}N. The second axis (20.6% of explained variance) was defined by a gradient of plant size, expressed in terms of plant height and volume. Interestingly, both whole-plant traits were negatively correlated with the peak of productivity, meaning that species with earlier phenology produced bigger plants. Plant volume and height were also positively correlated with leaf size and SRA, respectively. Finally, the third axis (13.98% of the total variance) was mainly associated with some morphological traits involved in the plant economics spectrum. Thus, those species that

invested less in leaf and root surface per unit of biomass (i.e. with lower values of SLA and SRA, respectively) developed thicker roots. Finally, seed mass was positively correlated with LNC and $\delta^{13}C$, and negatively with $\delta^{15}N$.

Supplementary Figure 2. A workflow of the study detailing the specific statistical analyses used for each of the objectives addressed on it.

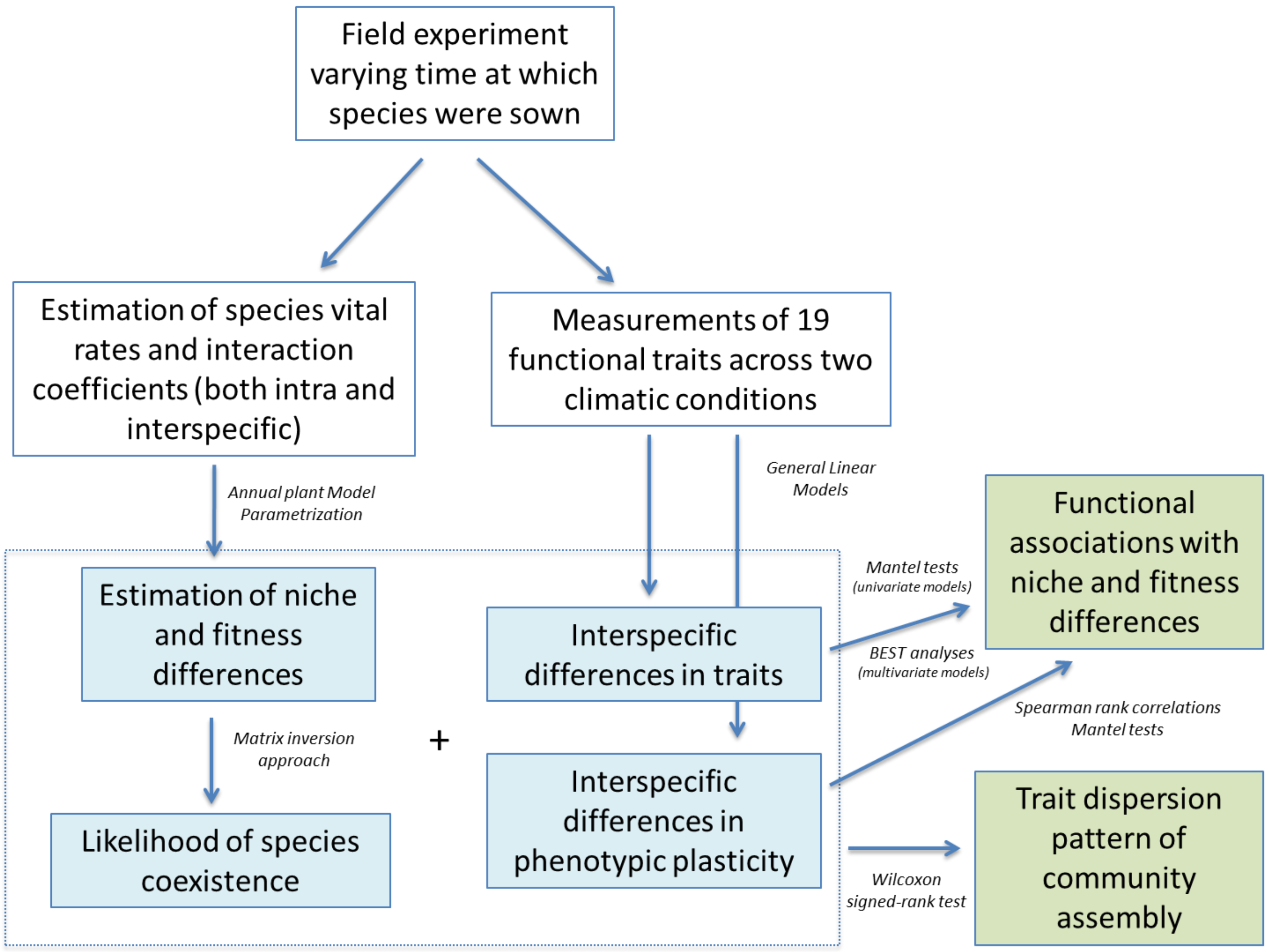

Supplement: Supplementary file 1 — Supplementary Information [file 41467_2019_10453_MOESM1_ESM.pdf]
